# Application of a deep learning system in glaucoma screening and further classification with colour fundus photographs: a case control study

**DOI:** 10.1186/s12886-022-02730-2

**Published:** 2022-12-12

**Authors:** Kuo-Hsuan Hung, Yu-Ching Kao, Yu-Hsuan Tang, Yi-Ting Chen, Chuen-Heng Wang, Yu-Chen Wang, Oscar Kuang-Sheng Lee

**Affiliations:** 1 Department of Ophthalmology, Chang-Gung Memorial Hospital, Linkou, No.5, Fu-Hsing St., Kuei Shan Hsiang, Tao Yuan Hsien, Taiwan; 2 Chang-Gung University College of Medicine, No.259 Wen-Hwa 1st Road, Kuei Shan Hsiang, Tao Yuan Hsien, Taiwan; 3 Institute of Clinical Medicine, National Yang Ming Chiao Tung University, No.201, Sec.2, Shih-Pai Rd. Peitou, R.O.C, Taipei, 112 Taiwan; 4 Muen Biomedical and Optoelectronics Technologies Inc., Taipei, Taiwan; 5 Stem Cell Research Centre, National Yang Ming Chiao Tung University, Taipei, Taiwan; 6 Department of Orthopedics, China Medical University Hospital, Taichung, Taiwan

**Keywords:** Glaucoma screening and classification, Deep learning system, Normal- tension glaucoma, Colour fundus photograph, High myopia

## Abstract

**Background:**

To verify efficacy of automatic screening and classification of glaucoma with deep learning system.

**Methods:**

A cross-sectional, retrospective study in a tertiary referral hospital. Patients with healthy optic disc, high-tension, or normal-tension glaucoma were enrolled. Complicated non-glaucomatous optic neuropathy was excluded. Colour and red-free fundus images were collected for development of DLS and comparison of their efficacy. The convolutional neural network with the pre-trained EfficientNet-b0 model was selected for machine learning. Glaucoma screening (Binary) and ternary classification with or without additional demographics (age, gender, high myopia) were evaluated, followed by creating confusion matrix and heatmaps. Area under receiver operating characteristic curve (AUC), accuracy, sensitivity, specificity, and F1 score were viewed as main outcome measures.

**Results:**

Two hundred and twenty-two cases (421 eyes) were enrolled, with 1851 images in total (1207 normal and 644 glaucomatous disc). Train set and test set were comprised of 1539 and 312 images, respectively. If demographics were not provided, AUC, accuracy, precision, sensitivity, F1 score, and specificity of our deep learning system in eye-based glaucoma screening were 0.98, 0.91, 0.86, 0.86, 0.86, and 0.94 in test set. Same outcome measures in eye-based ternary classification without demographic data were 0.94, 0.87, 0.87, 0.87, 0.87, and 0.94 in our test set, respectively. Adding demographics has no significant impact on efficacy, but establishing a linkage between eyes and images is helpful for a better performance. Confusion matrix and heatmaps suggested that retinal lesions and quality of photographs could affect classification. Colour fundus images play a major role in glaucoma classification, compared to red-free fundus images.

**Conclusions:**

Promising results with high AUC and specificity were shown in distinguishing normal optic nerve from glaucomatous fundus images and doing further classification.

**Supplementary Information:**

The online version contains supplementary material available at 10.1186/s12886-022-02730-2.

## Background

Glaucoma is one of the leading causes of blindness worldwide, affecting quality of life and working ability if diagnosis is delayed [1]. Glaucoma usually develops in elder people, presenting glaucomatous optic neuropathy (GON), corresponding retinal nerve fibre layer (RNFL) and visual field (VF) defects [2]. Since early symptoms could be insidious, effective glaucoma screening is important for early diagnosis, especially in health professional shortage areas.

Heidelberg retinal tomography (HRT), optical coherence tomography (OCT), VF tests, and colour fundus photography with red-free imaging, are pivotal armamentarium for glaucoma diagnosis [3]. Although HRT and OCT can detect changes of the optic disc and surrounding RNFL, quality of images and availability of facilities limit their wide application. By contrast, fundus imaging is easily equipped, less technique-dependant, and already widely used, which is reasonable to be a candidate facility for glaucoma screening. With red-free imaging, disc haemorrhage (DH) and wedge-shaped RNFL defects can be easily detected as clues of glaucoma; however, its value in DLS-facilitated glaucoma screening and classification is required to be explored.

Artificial intelligence (A.I.) with deep learning system (DLS) has widely been explored in ophthalmology for screening diabetic retinopathy (DR), macular degeneration, papilledema, and glaucoma [4–7]. Compared to commercialised products in detecting DR, DLS for glaucoma screening and classification is still under development. OCT scanning for RNFL thickness or combined with fundus images in presented various efficacy of glaucoma diagnosis and predicting progression with area under receiver operating characteristic curve (AUC) from 83 to 96% [8–10]. When detecting glaucoma with fundus images from referred diabetic patients, AUC of 94.2%, sensitivity of 96.4%, and specificity of 87.2% were found, respectively [11]. Efficacy of fundus imaging-based DLS showed that AUC, sensitivity, and specificity were 98.6%, 95.6%, and 92.0%, respectively in detection of GON [7]. When equipped with different image-cropping ratio on optic nerve head (ONH) or peripheral images in DLS, the results revealed that information from ONH and surrounding retina both contributed to glaucoma diagnosis [12]. With the pre-trained algorithm, even fundus photographs from smartphones can also be considered as an interface to screen glaucoma, which revealed better performance in advanced stage [13].

Besides glaucoma screening, glaucoma progression in myopic cohort with normal- tension glaucoma (NTG) had also been verified with machine learning [14]. Different from high-tension glaucoma (HTG), NTG is possibly overlooked due to its normal intraocular pressure (IOP) and requirements of mandatory ocular examinations and systemic survey to exclude other optic neuropathy before diagnosis. In published articles, DLS can reach an AUC of 0.966 in detecting structural changes with OCT-based parameters between glaucoma suspects and early NTG patients [15]. Although DH in fundus imaging is one common presentation of NTG, whether other phenotypes exist in fundus images to distinguish NTG from other types of glaucoma is not fully explored. Since different algorithms, enrolled parameters, and results exist between DLSs, we aimed to develop DLS for glaucoma screening and classification in this study.

## Methods

### Patient

The study was approved by the Institutional Review Board of Chang Gung Memorial Hospital, Linkou (No.201801801B0C601) and adhered to the tenets of the Declaration of Helsinki. Informed consent was waived in all patients and all images were turned into anonymous information before training and testing. Diagnosis and enrolment of glaucoma patients was based on Anderson’s VF criteria. In brief, a vertically enlarged cupping, defect of RNFL in colour/red-free fundus images/OCT, and glaucomatous VF defect were documented to confirm glaucoma diagnosis. At least two consistent glaucomatous VF defects were recorded as baseline data before diagnosis, except for end-stage glaucoma patients with prominent clinical presentation and imaging findings, such as total cupping, pale disc, elevated IOP, and tunnel vision. Patients with HTG, NTG, and non-GON were enrolled. Among GON patients, those with IOP equal or higher than 22 mmHg were diagnosed as HTG. Treatment-naïve patients with long-term IOP equal or lower than 21 mmHg were viewed as NTG. Pre-perimetric glaucoma and glaucoma suspects were not enrolled.

Fundus images were taken with fundus cameras (Carl Zeiss VISUCAM 524, Canon CR-2AF, and KOWA nonmyd 8 s). The colour fundus photographs and red-free fundus images were taken in two ways, optic nerve head-centred and papillo-macular area-centred images. Although three machines for photography were used with different resolution, all enrolled images were resized into the same resolution before analysis. Demographics, including age, gender, high myopia and diagnosis, were collected. High myopia was defined as spherical equivalent equal or less than -6 D or axial length longer than 26 mm. All the fundus images were designated to train or test set. At first, we dispatched images to the train or test set based on the patients; therefore, same patient would not appear in the train and test set at the same time. Then, we further divided the train set into training and validation set based on eye level, which meant images from the same eye would be fully partitioned into either training or validation set.

We trained the DLS by using AutoDL API (Application Programming Interface), which is the API of MAIA software (Medical Artificial Intelligence Aggregator) (Muen Biomedical and Optoelectronic Technologist, Inc, Taipei city, Taiwan). We applied the convolutional neural network (CNN) with the model structure of EfficientNet-b0 pre-trained on ImageNet [16, 17]. All fundus images were resized to 256*256. Data augmentations and dropout layers were applied to prevent overfitting [18]. Then, the extracted feature maps from CNN were flattened and concatenated with demographic features, which was inputted into the fully connected layers. The training epoch was 100, and the batch size was 32. The loss function was cross-entropy loss, and the optimizer was Adam [19]. During the training process, the learning rate was scheduled by a one-cycle of cosine annealing strategy [20, 21]. Five-fold cross validation was performed to validate the models. Among the five models from fivefold cross validation, the one with the highest F1 score was chosen for model testing. In binary classification, images were classified into GON or non-GON by DLS with or without demographics. Similarly, in ternary classification, non-GON people, HTG, and NTG were classified with or without demographic data. Confusion maps and heatmaps were created after analysis.

AUC, accuracy, precision, sensitivity, specificity, and F1 score were used as outcome measures. Precision (positive predictive value) was defined as the fraction of true glaucoma among all pictures classified as glaucoma. F1 score was selected to evaluate the performance of model prediction. SPSS statistics software was used to calculate *p* value and other statistics. *P* value < 0.05 was viewed as statistically significant. The independent t test and the Chi-squared test were used to compare data in binary classification. One-way analysis of variance (ANOVA) with Tukey’s honestly significant difference (HSD) test and the Chi-squared test were utilized to compare data in ternary classification and between combinations of demographic data.

## Results

Two hundred and twenty-two cases (421 eyes) were enrolled, half male and half female, with 1851 raw images in sum. Among 421 eyes, 290 eyes presented healthy optic nerves and the rest 131 eyes had GON, of which 85 eyes were HTG and the other 46 eyes had long-term normal IOP.

In the binary classification, 1207 raw images of the optic disc were non-GON, and 644 images were GON. In ternary classification, 644 images of GON were further classified into 235 images of NTG and 409 images of HTG. There were 1851 images included in the dataset, in which 1231 images (283 eyes) were used as a training set and 308 images (68 eyes) were dispatched to a validation set. The rest 312 images (70 eyes) were prepared as a test set. Mean age of our healthy and GON patients in binary classification were 48.33 ± 18.54 and 61.22 ± 16.79 years, respectively, with significant difference (*p* < 0.001). In Chi-squared test, there was no difference between glaucoma and control group in gender (*p* = 0.49). In ternary classification, mean age of non-GON, NTG, and HTG patients were 48.33 ± 18.54, 60.1 ± 17.85, and 61.87 ± 16.28, respectively. *P* value < 0.001 was noted in ANOVA test, which meant three groups have significant difference in age distribution. Demographic data were shown in Table 1.

**Table 1:** Demographic data of healthy people, NTG, and HTG patients

features	Healthy (*n* = 165)	NTG (*n* = 30)	HTG (*n* = 52)	*P* value
Age (years)	48.33 ± 18.54	60.1 ± 17.85	61.87 ± 16.28	< 0.001^*^
Gender (female)	50.9%	50%	44.2%	0.04^†^
High myopia	16.3%	20%	19.2%	

*NTG* Normal-tension glaucoma, *HTG* High-tension glaucoma

^*^One-way ANOVA

^†^X^2^ test

**Fig. 1:**
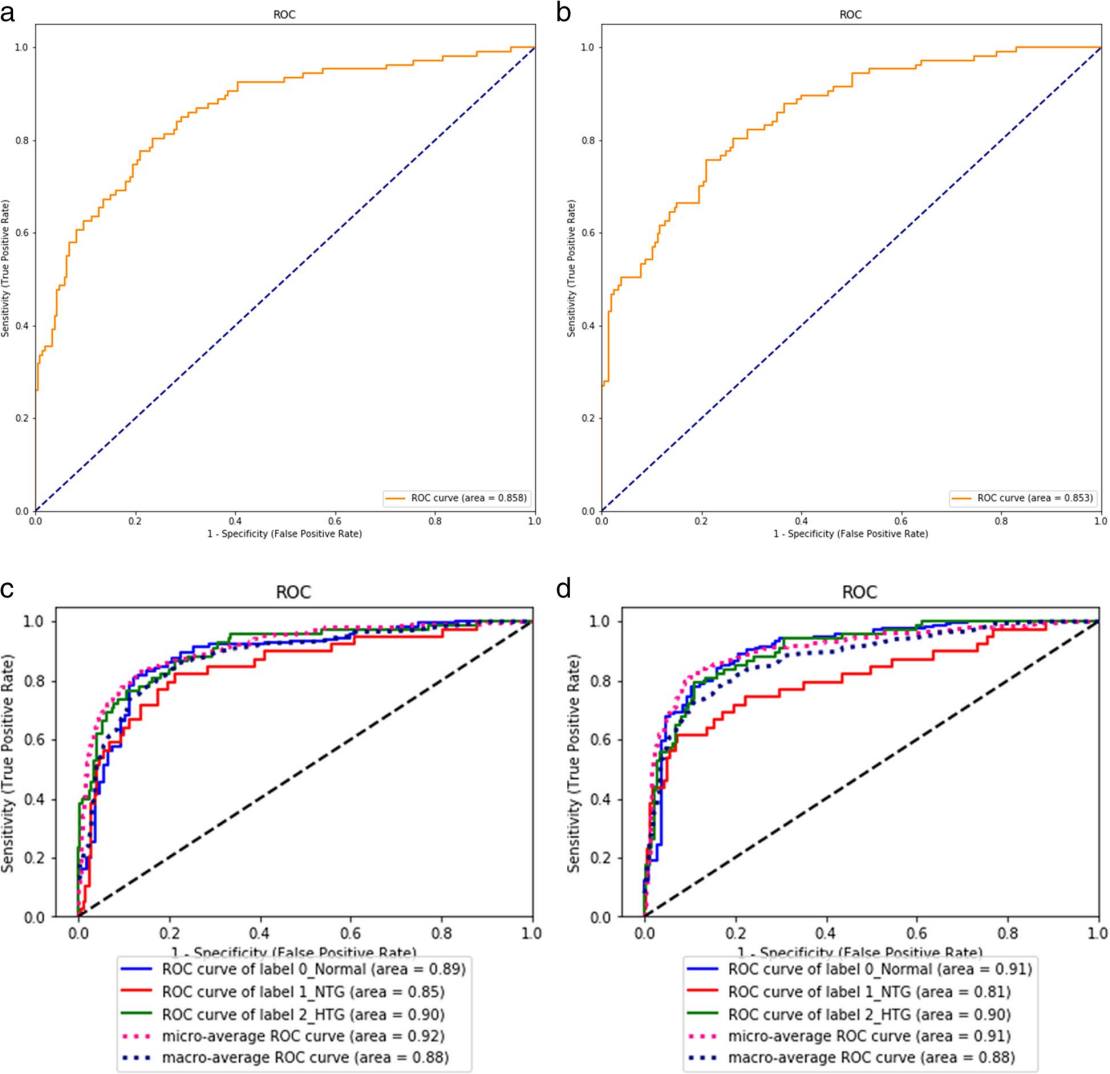
The ROC curves with AUC in binary and ternary classification with or without demographics. Binary classification with (**a**) and without (**b**) information of age, gender, and high myopia in test set. Ternary classification with (**c**) and without (**d**) information of age, gender, and high myopia in test set

**Fig. 2:**
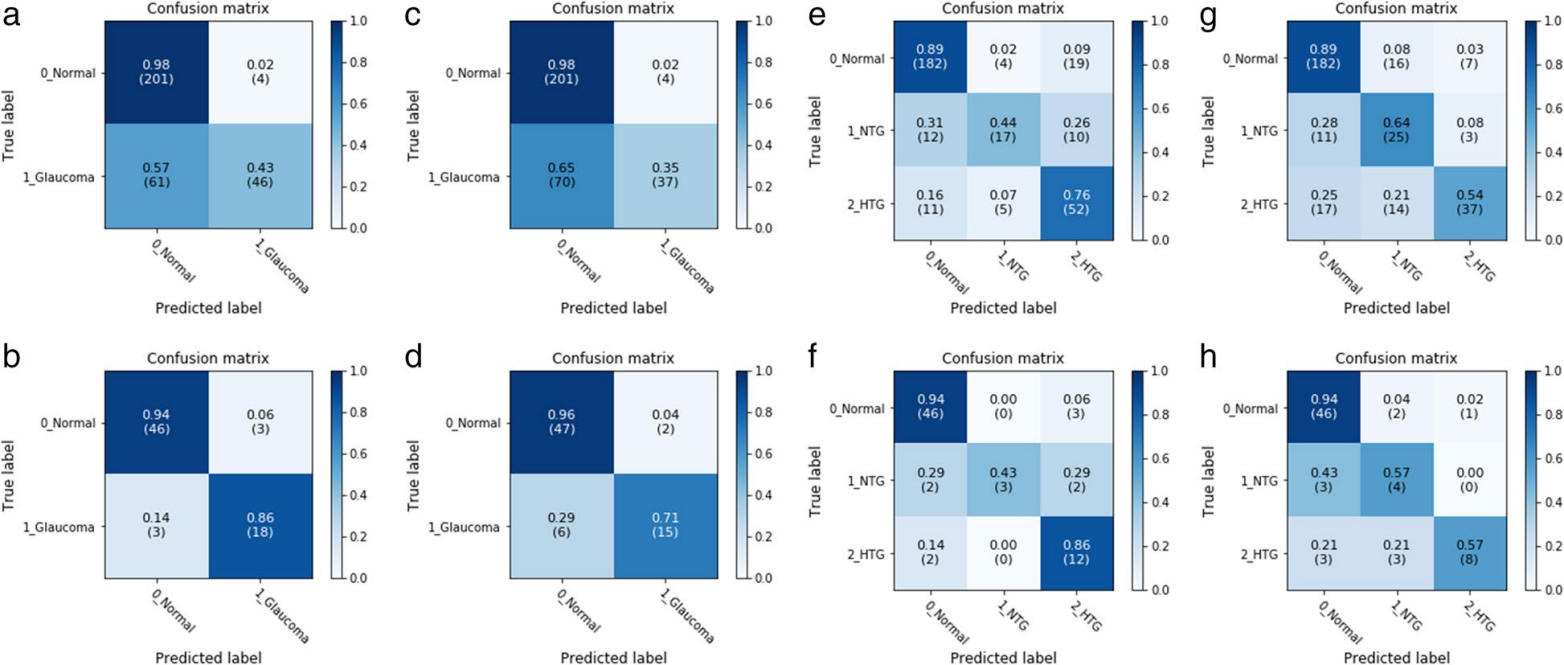
Image- or eye-based confusion matrix in test set of binary and ternary classification. Image- (**a**) and eye-based (**b**) analysis of binary classification (0.0 = normal; 1.0 = glaucoma) in test set. After adding information of age, gender, and high myopia, results of binary classification in test set with image- (**c**) or eye-based (**d**) analysis. Image- (**e**) and eye-based (**f**) analysis of ternary classification (0.0 = normal; 1.0 = normal-tension glaucoma; 2.0 = high-tension glaucoma) in test set. After adding information of age, gender, and high myopia, results of ternary classification in test set with image- (**g**) or eye-based (**h**) analysis

**Fig. 3:**
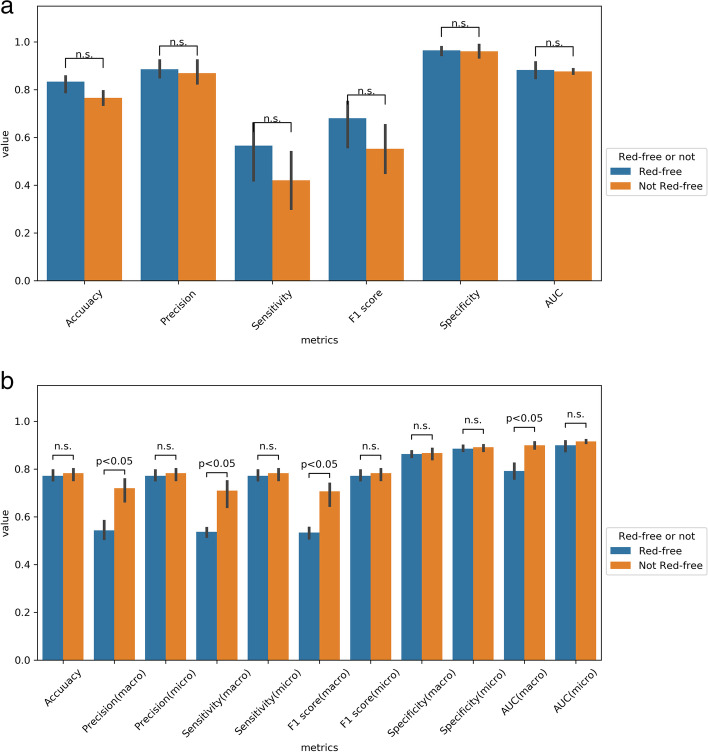
The outcome measures in image-based analysis of red-free or colour fundus images. Test metrics calculated from red-free fundus images and colour fundus images were compared by paired t-test. In binary classification, red-free fundus images achieved better performance in number, which was not statistically significant (**a**). Colour fundus images achieved better performance in ternary classification, in which statistically significant differences were observed (**b**). n.s. = not statistically significant

**Fig. 4:**
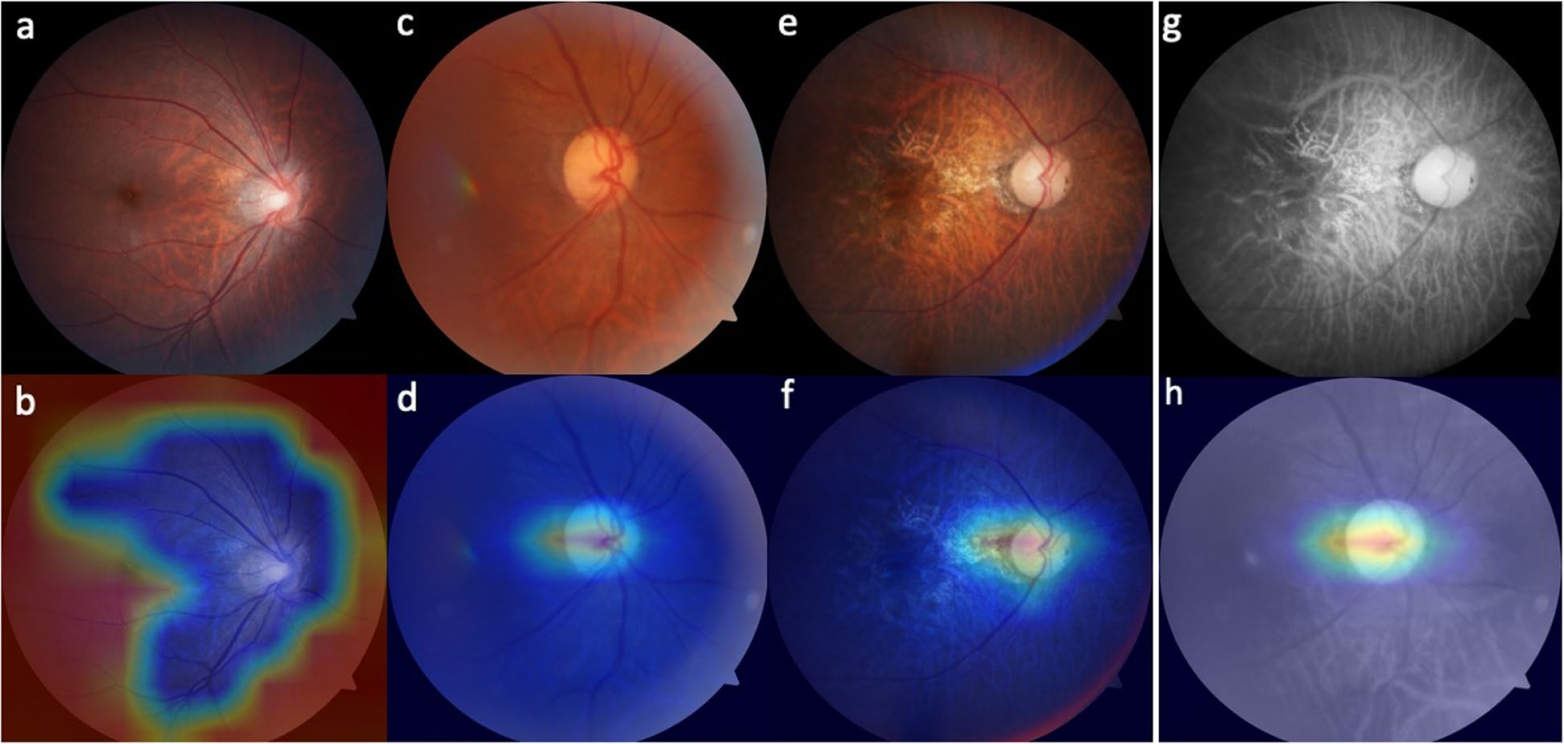
Binary classification presented by heatmap to show weighted area of deep learning system. Non-glaucomatous fundus (**a**) showed a weighted area peripherally, outside optic disc (**b**). Weighted area presented temporal to optic disc in normal-tension glaucoma (**c** and **d**) and high-tension glaucoma (**e** and **f**) in binary classification. Red-free fundal picture and its associated heatmap (**g** and **h**) in our study

Our model was verified in two ways, including image- or eye-based analysis. Each image was used as one independent data in the former analysis; while, images from the same eye was annotated beforehand as a specific parameter for later analysis. The results in different analyses were presented in Tables 2 and 3. Five-fold cross validation were performed with no significantly different result. In brief, precision, accuracy, sensitivity, specificity, F1 score, and AUC in image-based glaucoma screening were 0.92, 0.79, 0.43, 0.98, 0.59, and 0.85 in test set. After providing the linkage between each image and the eye, the eye was classified as glaucoma if any of its images was predicted as positive. In this eye-based analysis, precision, accuracy, sensitivity, specificity, F1 score and AUC were 0.86, 0.91, 0.86, 0.94, 0.86, and 0.98 in test set, in which accuracy, sensitivity, F1 score, and AUC were largely improved, while precision and specificity slightly decreased. The receiver operating characteristic curves (ROC curves) in binary classification with or without demographic information in test set were shown in Fig. 1 (a and b). Confusion matrix to present image- or eye- based binary classification in test set was shown in Fig. 2 (a and b). Confusion matrix of binary classification after adding extra information was shown in Fig. 2 (c and d). We added information about age, gender, and high myopia into our model, no improvement was observed in the outcome measures in both validation and test set in binary classification (Tables 2 and 3). When comparing the outcome measures between red-free and colour fundus images, red-free imaging showed higher efficacy in most parameters in glaucoma screening, but not reached statistical significance (Tables 4 and 5, Fig. 3a). In the heatmaps of binary classification, a weighted area was found outside non-GON optic disc at four quadrants (Fig. 4 a and b). A weighted area temporal to the optic disc (Fig. 4 c to h) was shown in the heatmaps of GON.

**Table 2:** Efficacy of binary and ternary classification by the deep learning system

	without additional information				with age and gender information			
	validation set		testing set		validation set		testing set	
Metrics	Image-based	Eye-based	Image-based	Eye-based	Image-based	Eye-based	Image-based	Eye-based
**Binary classification**								
Accuracy	0.82(0.03)	0.88(0.04)	0.79	0.91	0.84(0.05)	0.89(0.06)	0.83	0.87
Precision	0.85(0.06)	0.78(0.07)	0.92	0.86	0.83(0.06)	0.79(0.07)	0.83	0.75
Sensitivity	0.61(0.12)	0.86(0.09)	0.43	0.86	0.69(0.09)	0.89(0.07)	0.63	0.86
Specificity	0.94(0.03)	0.89(0.04)	0.98	0.94	0.92(0.05)	0.88(0.06)	0.93	0.88
F1 score	0.70(0.06)	0.82(0.06)	0.59	0.86	0.75(0.08)	0.84(0.06)	0.71	0.8
AUC	0.91(0.02)	0.99(0.01)	0.85	0.98	0.91(0.04)	0.98(0.01)	0.9	0.98
**Ternary classification**								
Accuracy	0.78(0.04)	0.82(0.03)	0.8	0.87	0.77(0.05)	0.81(0.05)	0.77	0.81
Precision (macro)	0.65(0.05)	0.69(0.07)	0.73	0.88	0.64(0.08)	0.64(0.09)	0.69	0.72
Precision (micro)	0.78(0.04)	0.82(0.03)	0.8	0.87	0.77(0.05)	0.81(0.05)	0.77	0.81
Sensitivity (macro)	0.66(0.06)	0.68(0.06)	0.7	0.74	0.63(0.06)	0.65(0.08)	0.69	0.72
Sensitivity (micro)	0.78(0.04)	0.82(0.03)	0.8	0.87	0.77(0.05)	0.81(0.05)	0.77	0.81
Specificity (macro)	0.87(0.03)	0.89(0.03)	0.88	0.91	0.86(0.03)	0.88(0.04)	0.87	0.89
Specificity (micro)	0.89(0.02)	0.91(0.02)	0.9	0.94	0.89(0.03)	0.91(0.03)	0.89	0.91
F1 score (macro)	0.65(0.05)	0.67(0.07)	0.7	0.77	0.62(0.07)	0.64(0.08)	0.69	0.72
F1 score (micro)	0.78(0.04)	0.82(0.03)	0.8	0.87	0.77(0.05)	0.81(0.05)	0.77	0.81
AUC (macro)	0.87(0.04)	0.91(0.04)	0.88	0.9	0.85(0.05)	0.90(0.04)	0.86	0.9
AUC (micro)	0.91(0.03)	0.93(0.02)	0.91	0.94	0.91(0.03)	0.94(0.02)	0.89	0.93

*AUC* Area under receiver operating characteristic curve

**Table 3:** Efficacy of binary and ternary classification with or without information of high myopia

	with information of high myopia only				with age, gender, and high myopia information			
	validation set		testing set		validation set		testing set	
Metrics	Image-based	Eye-based	Image-based	Eye-based	Image-based	Eye-based	Image-based	Eye-based
**Binary classification**								
Accuracy	0.81(0.05)	0.88(0.04)	0.82	0.86	0.81(0.02)	0.88(0.06)	0.76	0.89
Precision	0.89(0.06)	0.86(0.06)	0.87	0.74	0.88(0.08)	0.86(0.10)	0.9	0.88
Sensitivity	0.52(0.18)	0.77(0.18)	0.57	0.81	0.53(0.10)	0.77(0.18)	0.35	0.71
Specificity	0.96(0.02)	0.94(0.03)	0.96	0.88	0.95(0.06)	0.92(0.09)	0.98	0.96
F1 score	0.63(0.16)	0.79(0.10)	0.69	0.77	0.65(0.06)	0.79(0.11)	0.5	0.79
AUC	0.89(0.04)	0.99(0.01)	0.89	0.98	0.90(0.02)	0.99(0.01)	0.86	0.98
**Ternary classification**								
Accuracy	0.76(0.03)	0.79(0.05)	0.75	0.86	0.75(0.04)	0.81(0.02)	0.78	0.83
Precision (macro)	0.63(0.04)	0.66(0.07)	0.61	0.73	0.63(0.04)	0.65(0.06)	0.7	0.74
Precision (micro)	0.76(0.03)	0.79(0.05)	0.75	0.86	0.75(0.04)	0.81(0.02)	0.78	0.83
Sensitivity (macro)	0.65(0.06)	0.67(0.09)	0.6	0.7	0.64(0.04)	0.68(0.06)	0.69	0.69
Sensitivity (micro)	0.76(0.03)	0.79(0.05)	0.75	0.86	0.75(0.04)	0.81(0.02)	0.78	0.83
Specificity (macro)	0.87(0.02)	0.88(0.03)	0.85	0.9	0.86(0.02)	0.89(0.02)	0.86	0.87
Specificity (micro)	0.88(0.02)	0.90(0.02)	0.87	0.93	0.88(0.02)	0.90(0.01)	0.89	0.91
F1 score (macro)	0.64(0.05)	0.66(0.07)	0.6	0.71	0.63(0.04)	0.66(0.05)	0.68	0.7
F1 score (micro)	0.76(0.03)	0.79(0.05)	0.75	0.86	0.75(0.04)	0.81(0.02)	0.78	0.83
AUC (macro)	0.85(0.04)	0.90(0.04)	0.87	0.9	0.87(0.05)	0.92(0.04)	0.88	0.91
AUC (micro)	0.89(0.04)	0.93(0.03)	0.91	0.95	0.90(0.04)	0.94(0.03)	0.92	0.95

*AUC* Area under receiver operating characteristic curve

**Table 4:** Efficacy of binary and ternary classification stratified by red-free photographs and non-red-free photographs

	without additional information				with age and gender information			
	validation set		testing set		validation set		testing set	
Metrics	Red-free	Not red-free	Red-free	Not red-free	Red-free	Not red-free	Red-free	Not red-free
**Binary classification**								
Accuracy	0.82(0.04)	0.82(0.03)	0.85	0.72	0.84(0.06)	0.85(0.07)	0.85	0.80
Precision	0.86(0.06)	0.85(0.10)	0.94	0.88	0.84(0.07)	0.82(0.10)	0.84	0.81
Sensitivity	0.66(0.10)	0.54(0.20)	0.59	0.26	0.72(0.07)	0.65(0.13)	0.69	0.57
Specificity	0.93(0.03)	0.94(0.05)	0.98	0.98	0.91(0.06)	0.93(0.05)	0.94	0.93
F1 score	0.74(0.06)	0.63(0.13)	0.73	0.41	0.77(0.07)	0.72(0.12)	0.76	0.67
AUC	0.92(0.03)	0.91(0.03)	0.87	0.86	0.91(0.05)	0.91(0.05)	0.92	0.88
**Ternary classification**								
Accuracy	0.77(0.06)	0.79(0.05)	0.81	0.80	0.77(0.07)	0.78(0.07)	0.76	0.79
Precision (macro)	0.64(0.07)	0.67(0.06)	0.53	0.77	0.59(0.08)	0.67(0.09)	0.50	0.73
Precision (micro)	0.77(0.06)	0.79(0.05)	0.81	0.80	0.77(0.07)	0.78(0.07)	0.76	0.79
Sensitivity (macro)	0.64(0.08)	0.68(0.07)	0.55	0.74	0.60(0.07)	0.66(0.08)	0.51	0.75
Sensitivity (micro)	0.77(0.06)	0.79(0.05)	0.81	0.80	0.77(0.07)	0.78(0.07)	0.76	0.79
Specificity (macro)	0.87(0.04)	0.87(0.03)	0.88	0.87	0.86(0.04)	0.86(0.04)	0.85	0.89
Specificity (micro)	0.89(0.03)	0.90(0.03)	0.90	0.90	0.88(0.04)	0.89(0.04)	0.88	0.89
F1 score (macro)	0.63(0.07)	0.66(0.06)	0.54	0.74	0.59(0.07)	0.64(0.09)	0.50	0.73
F1 score (micro)	0.77(0.06)	0.79(0.05)	0.81	0.80	0.77(0.07)	0.78(0.07)	0.76	0.79
AUC (macro)	0.87(0.05)	0.88(0.04)	0.77	0.92	0.84(0.06)	0.87(0.05)	0.75	0.90
AUC (micro)	0.90(0.04)	0.91(0.04)	0.91	0.92	0.90(0.04)	0.91(0.04)	0.87	0.91

*AUC* Area under receiver operating characteristic curve

**Table 5:** Efficacy of binary and ternary classification with or without information of high myopia stratified by red-free photographs and non-red-free photographs

	with information of high myopia only				with age, gender, and high myopia information			
	validation set		testing set		validation set		testing set	
Metrics	Red-free	Not red-free	Red-free	Not red-free	Red-free	Not red-free	Red-free	Not red-free
**Binary classification**								
Accuracy	0.80(0.05)	0.81(0.08)	0.85	0.79	0.81(0.02)	0.81(0.03)	0.77	0.76
Precision	0.91(0.07)	0.88(0.11)	0.89	0.84	0.89(0.10)	0.86(0.10)	0.86	0.95
Sensitivity	0.55(0.16)	0.47(0.24)	0.63	0.51	0.57(0.11)	0.46(0.12)	0.35	0.34
Specificity	0.96(0.03)	0.97(0.03)	0.96	0.95	0.94(0.08)	0.96(0.04)	0.97	0.99
F1 score	0.67(0.12)	0.57(0.26)	0.74	0.64	0.69(0.06)	0.58(0.08)	0.50	0.50
AUC	0.89(0.04)	0.90(0.03)	0.90	0.87	0.91(0.03)	0.89(0.03)	0.83	0.89
**Ternary classification**								
Accuracy	0.74(0.06)	0.78(0.04)	0.75	0.74	0.73(0.05)	0.78(0.04)	0.77	0.80
Precision (macro)	0.61(0.05)	0.66(0.04)	0.55	0.64	0.61(0.05)	0.64(0.04)	0.60	0.74
Precision (micro)	0.74(0.06)	0.78(0.04)	0.75	0.74	0.73(0.05)	0.78(0.04)	0.77	0.80
Sensitivity (macro)	0.63(0.07)	0.67(0.08)	0.53	0.61	0.61(0.06)	0.66(0.04)	0.56	0.73
Sensitivity (micro)	0.74(0.06)	0.78(0.04)	0.75	0.74	0.73(0.05)	0.78(0.04)	0.77	0.80
Specificity (macro)	0.86(0.03)	0.86(0.04)	0.87	0.83	0.86(0.03)	0.86(0.02)	0.85	0.87
Specificity (micro)	0.87(0.03)	0.89(0.02)	0.88	0.87	0.87(0.03)	0.89(0.02)	0.88	0.90
F1 score (macro)	0.61(0.06)	0.66(0.06)	0.54	0.62	0.61(0.05)	0.65(0.04)	0.56	0.73
F1 score (micro)	0.74(0.06)	0.78(0.04)	0.75	0.74	0.73(0.05)	0.78(0.04)	0.77	0.80
AUC (macro)	0.84(0.06)	0.87(0.03)	0.83	0.88	0.87(0.07)	0.88(0.04)	0.82	0.90
AUC (micro)	0.87(0.06)	0.90(0.03)	0.92	0.91	0.89(0.05)	0.92(0.03)	0.91	0.92

*AUC* Area under receiver operating characteristic curve

**Fig. 5:**
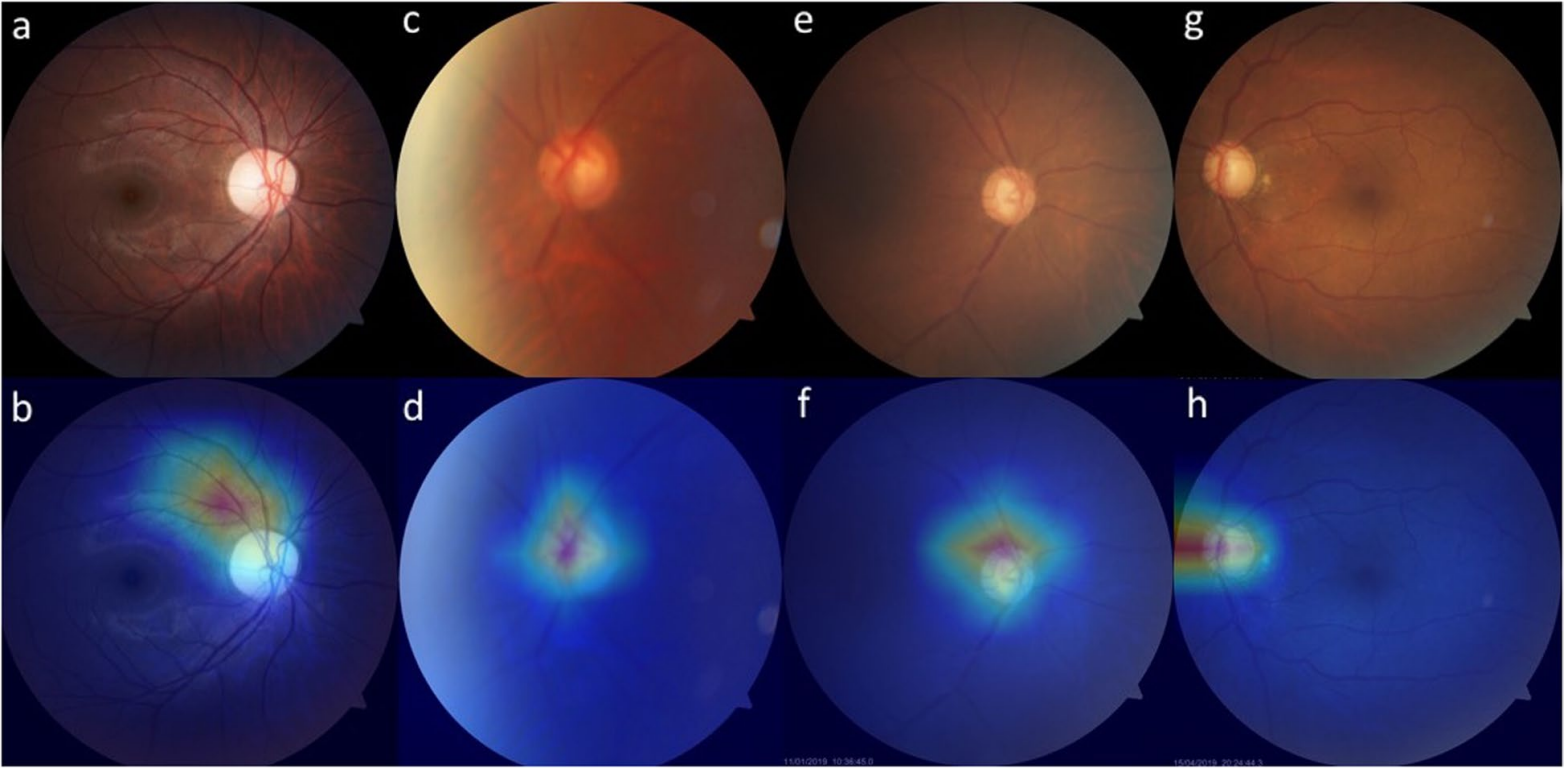
Ternary classification presented by heatmap to show weighted area of deep learning system. Non-glaucomatous fundus (**a**) showed weighted area supero-temporal to optic disc (**b**). Weighted area presented at optic disc in high-tension glaucoma (**c** and **d**) in ternary classification. Misclassification of normal-tension glaucoma into high-tension glaucoma (**e**), showing weighted area nasal to optic disc (**f**) in the left eye. High-tension glaucoma was misclassified into normal-tension glaucoma (**g**), presenting a weighted area inferior to optic disc (**h**) in the right eye

To verify DLS in ternary classification, validation set and test set with or without demographics were analyzed. The results in different sets were presented in Tables 2 and 3. To provide prediction without demographics in an eye-based manner, we averaged the predicted probabilities of each image. In this eye-based analysis of test set in ternary classification, all outcome metrics were improved, achieving an accuracy of 0.87, F1 score(macro) of 0.77, and AUC(macro) of 0.9. The ROC curves in ternary classification with or without demographics in test set were shown in Fig. 1 (c and d). Confusion matrix of ternary classification without demographics in test set was shown in Fig. 2 (e and f). Distribution of our results of ternary classification after adding clinical information was shown in Fig. 2 g and h. No remarkable increase of all the outcome measures was noted after adding extra information into image- and eye-based analysis in ternary classification (Tables 2 and 3). We compared the outcome measures of red-free and colour fundus images, colour fundus images had a better performance in ternary classification with statistically significant difference (Tables 4 and 5, Fig. 3b),

The results of ternary classification were also visualized in heatmaps, within which a weighted area was mainly supero-temporal to normal disc (Fig. 5 a and b). Heatmaps of the eyes with HTG showed a weighted area over the disc (Fig. 5 c and d). However, heatmaps of NTG presented a weighted area superior to the disc (Fig. 5 e and f). Examples of misclassification on heatmap in ternary classification were shown in Fig. 5 g and h.

## Discussion

In this study, an image-based or eye-based DLS was developed to perform glaucoma screening. Moreover, an algorithm was developed to verify ternary classification for non-GON, HTG, and NTG patients. Although we only enrolled 222 patients (421 eyes) with 1851 images, in image-based analysis of binary classification, AUC reached 0.85 in test set with the assistance of dropout function and data augmentation. In eye-based analysis, accuracy was improved from 0.79 to 0.91 and F1 score had achieved 0.86. In ternary classification, F1 score(macro) achieved 0.77, and AUC reached 0.9 in eye-based analysis. Confusion matrix and heatmap provided us more details about distribution of data after classification and weighted area in DLS.

Although age, gender, and myopia are viewed as risk factors for open angle- or angle closure glaucoma [22–24], no remarkable improvement of performance has been found in our binary and ternary classification when providing demographics. In clinical settings, these factors are used to evaluate glaucoma suspect; however, it seems that image-only DLS is capable of doing screening and classification without additional information. Furthermore, impacts of age, gender, and myopia on the eye are fundamentally based on theories that aging oxidative stress to trabecular meshwork, structural change at the angle of anterior chamber, and circulation changes around optic nerve head. Other complicated influences of high myopia, such as peripapillary atrophy, retinal thinning, and tilted optic disc, also potentially play a role in glaucoma development. These molecular and structural changes may leave no discriminative clues in fundus images, resulting in less impacts in our results. Consequently, a simple fundus images-based screening system without demographics can be applied in telemedicine for fast screening.

Images of optic disc, OCT, VF, and clinical demographics had ever been chosen to verify the efficacy of glaucoma diagnosis with different algorithms in published studies. Li et al. evaluated efficacy of the DLS in detecting referable GON based on 70,000 colour fundus images alone from online dataset, presenting an AUC of 98.6%, sensitivity of 95.6%, and specificity of 92.0% [7]. Compared to their study, convincing result of our glaucoma screening was shown with an AUC of 98.0%, sensitivity of 86.0% and specificity of 94.0%, based on less images. Different methods of image extraction had also been integrated in fundus image-based DLSs, such as wavelet feature [25], features of ONH [26], and adaptive threshold-based image processing [27], in which the optic disc and RNFL were specifically segmented and extracted for analysis. However, misalignment and misclassification tend to develop when segmentation and localization fail to be synchronized. Since informative data exist in both optic nerve and the retina in glaucoma screening [9], in our study, we enrolled whole fundus images, including macula-centred, optic nerve head-centred, and red-free images to avoid overmanipulating data.

The advantage of our method is that it keeps most information within fundus images, explores the ability of DLSs, simulates the real-world clinical situation, and can be applied in daily practice. The disadvantage of analyzing the whole fundal pictures results from possible noise of any retinal or optic disc lesions and artifacts in images. When comparing the performance of DLS in binary and ternary classification with red-free and colour fundus images, red-free imaging seemed helpful in glaucoma screening but presented no statistical significance in our results. However, colour fundus images showed better and statistically significant performance in ternary classification. The sharper signal along RNFL defects in red-free imaging, compared to colour fundus images, may explain remarkable outcome measures in glaucoma screening and in clinical practice. However, indistinguishable RNFL defects may exist between HTG and NTG; therefore, colour images with more digital information are favoured in ternary classification. To mix two types of images for the algorithm maintains benefits of each component, but colour fundus images seem sufficient to help glaucoma screening and classification in DLS. Conclusion has not yet been made in which kind of images are suitable for DLS. How to balance pros and cons between maintaining enough amount of information and minimizing noise in images remains to be declared.

Although demographics seemed to play less role in our DLS for glaucoma diagnosis and classification, linkage between images and the eyes showed meaningful impacts on performance. In glaucoma screening, eye-based analysis improved all the outcome measures, compared to image-based analysis, except for precision and specificity. This phenomenon may be attributed to increased false positive since glaucoma is diagnosed when one of the images from the same eye is predicted to be positive. By contrast, the strategy averaging probability of all images to predict a final diagnosis was used in our ternary classification. This strategy improved all outcome measures in ternary classification.

According to the confusion matrixes (Fig. 2e-h), DLS for ternary classification is still effective to identify non-GON from GON, but less effective in identifying NTG from non-GON and HTG. This result may be attributed to several potential reasons, including small number of NTG eyes and natural entity of HTG/NTG that no remarkable morphological difference exists between their fundus images. By providing linkage between eyes and images, the performance can be improved in all outcome metrics. Moreover, performance can also be improved by using macro or micro averages when doing ternary classification. To further interpret confusion matrix, specificity was not significantly improved by adding demographics in both binary and ternary classification; meanwhile, this additional information did not remarkably improve classification of glaucoma. Similar to our multiple classification, one study aimed to identify GON with individual mean deviation in VF report from healthy people by stereo fundus images. Their results showed AUC from 0.89 to 0.97, according to different conditions [28]. Interestingly, performance of IOP prediction between a multivariate linear regression model (MLM) with 35 systemic variables and a DLS with colour fundus images showed that the former had a better predictive value [29]. The results may support that it may be better to use demographics to predict physiological parameters than to do glaucoma screening with images.

Heatmaps were used to visualize the viewpoint of the DLS. In binary classification, weighted area presented at peripheral retina in non-GON eyes and at the optic nerve in eyes with GON, presenting a different but efficient way for DLS to quickly identify glaucoma. Although DH or RNFL defect already existed in images, those GON misinterpreted into healthy optic disc may be resulted from artifact or other coexisting retinal lesions, such as macular pucker, myopic tessellated fundus, and large peripapillary atrophy (PPA), which showed that abnormal retinal presentations were first focused by DLS. Some glaucomatous images from the same eye were misinterpreted into non-GON at first; however, sensitivity from these data improved when linkage between images and the eye was built. Images of healthy optic disc that are misinterpreted into GON may be resulted from influence of tortuous vessel, underexposure area, and PPA in fundal images.

The heatmap in ternary classification still showed a weighted area at the optic disc in HTG group. HTG images misinterpreted into NTG presented a weighted area over vascular bifurcation, arteriovenous nicking, or nasal retina. Similar to the heatmaps in binary classification, lesions of retina or optic disc such as disc hemorrhage could mislead DLS to a wrong classification, even though remarkable RNFL defect existed at the same time. Different from heatmaps in binary classification, a weighted area presented at the region supero-temporal to the healthy optic nerve in the ternary classification. This phenomenon showed that DLS used different strategy to analyze data in binary and ternary classification.

The limitations of our study include limited case numbers, lack of remarkable retinal or optic disc lesions other than glaucoma, single ethnic background, and exclusion of pre-perimetric glaucoma. Small number of training and validation sets was viewed as a drawback in machine learning, which may affect accuracy of glaucoma screening and lead to overfitting [4]. However, dropout function, data augmentation, and analysis at eye level were used to achieve applicable accuracy and AUC in glaucoma screening and classification. Glaucoma screening in combined ocular diseases and detection of pre-perimetric glaucoma are still major challenges for DLS.

## Conclusions

Identification of glaucoma and further classification into high-tension and normal- tension glaucoma can be achieved with the assistance of DLS, especially at eye level. Although DLS with red-free fundus images can fulfill the purpose of glaucoma screening, DLS with colour fundus images showed a better result in glaucoma classification. Clinical demographics seem to show no remarkable impact on the outcome measures in the study.

## Supplementary Information

**Additional file 1: Supplementary Table 1.** Cross validation results of binary classifications.

**Additional file 2: Supplementary Table 2.** Cross validation results of trinary classifications.

**Additional file 3: Supplementary Table 3.** Cross validation results of binary classifications stratified by red-free photographs and non-red-free photographs.

**Additional file 4: Supplementary Table 4.** Cross validation results of trinary classifications stratified by red-free photographs and non-red-free photographs.

## Data Availability

The datasets generated or analysed during the current study are not publicly available due to the institutional regulations but are available from the corresponding author on reasonable request.

## Abbreviations

A.I.: Artificial intelligence
ANOVA: Analysis of variance
AUC: Area under receiver operating characteristic curve
CNN: Convolutional neural network
DH: Disc haemorrhage
DLS: Deep learning system
DR: Diabetic retinopathy
GON: Glaucomatous optic neuropathy
HRT: Heidelberg retinal tomography
HSD: Honestly significant difference
HTG: High-tension glaucoma
NTG: Normal-tension glaucoma
OCT: Optical coherence tomography
ONH: Optic nerve head
PPA: Peripapillary atrophy
RNFL: Retinal nerve fibre layer
ROC curves: Receiver operating characteristic curves
VF: Visual field
