# Supplementary material for: Application of a deep learning system in glaucoma screening and further classification with colour fundus photographs: a case control study
Source: BMC Ophthalmol. 2022 Dec 12;22:483. doi: 10.1186/s12886-022-02730-2 (PMC9743575; doi:10.1186/s12886-022-02730-2)
Supplement: Supplementary file 2 — Additional file 2: Supplementary Table 2. Cross validation results of trinary classifications. [file 12886_2022_2730_MOESM2_ESM.docx]

Supplementary Table 2. Cross validation results of trinary classifications

| Metrics | Image-based | | | | |  | Eye-based | | | | |
| --- | --- | --- | --- | --- | --- | --- | --- | --- | --- | --- | --- |
|  | Fold1 | Fold2 | Fold3 | Fold4 | Fold5 |  | Fold1 | Fold2 | Fold3 | Fold4 | Fold5 |
| **without additional information** | | | | | | | | | | | |
| Accuracy | 0.83 | 0.76 | 0.80 | 0.79 | 0.72 |  | 0.87 | 0.81 | 0.83 | 0.83 | 0.77 |
| Precision  (macro) | 0.72 | 0.67 | 0.68 | 0.61 | 0.59 |  | 0.81 | 0.73 | 0.63 | 0.63 | 0.66 |
| Precision  (micro) | 0.83 | 0.76 | 0.80 | 0.79 | 0.72 |  | 0.87 | 0.81 | 0.83 | 0.83 | 0.77 |
| Sensitivity  (macro) | 0.72 | 0.69 | 0.69 | 0.63 | 0.55 |  | 0.78 | 0.69 | 0.68 | 0.67 | 0.58 |
| Sensitivity  (micro) | 0.83 | 0.76 | 0.80 | 0.79 | 0.72 |  | 0.87 | 0.81 | 0.83 | 0.83 | 0.77 |
| Specificity  (macro) | 0.90 | 0.85 | 0.88 | 0.88 | 0.83 |  | 0.92 | 0.86 | 0.91 | 0.90 | 0.85 |
| Specificity  (micro) | 0.92 | 0.88 | 0.90 | 0.89 | 0.86 |  | 0.94 | 0.91 | 0.91 | 0.92 | 0.89 |
| F1 score  (macro) | 0.72 | 0.67 | 0.67 | 0.62 | 0.56 |  | 0.78 | 0.70 | 0.64 | 0.64 | 0.59 |
| F1 score (micro) | 0.83 | 0.76 | 0.80 | 0.79 | 0.72 |  | 0.87 | 0.81 | 0.83 | 0.83 | 0.77 |
| AUC (macro) | 0.92 | 0.87 | 0.90 | 0.86 | 0.81 |  | 0.96 | 0.89 | 0.95 | 0.90 | 0.86 |
| AUC (micro) | 0.94 | 0.90 | 0.93 | 0.91 | 0.86 |  | 0.96 | 0.92 | 0.96 | 0.93 | 0.90 |
| **with age and gender information** | | | | | | | | | | | |
| Accuracy | 0.79 | 0.72 | 0.83 | 0.82 | 0.70 |  | 0.83 | 0.76 | 0.89 | 0.85 | 0.74 |
| Precision  (macro) | 0.65 | 0.56 | 0.74 | 0.73 | 0.54 |  | 0.70 | 0.60 | 0.77 | 0.54 | 0.57 |
| Precision  (micro) | 0.79 | 0.72 | 0.83 | 0.82 | 0.70 |  | 0.83 | 0.76 | 0.89 | 0.85 | 0.74 |
| Sensitivity  (macro) | 0.66 | 0.59 | 0.72 | 0.63 | 0.54 |  | 0.70 | 0.59 | 0.77 | 0.61 | 0.56 |
| Sensitivity  (micro) | 0.79 | 0.72 | 0.83 | 0.82 | 0.70 |  | 0.83 | 0.76 | 0.89 | 0.85 | 0.74 |
| Specificity  (macro) | 0.88 | 0.83 | 0.89 | 0.88 | 0.82 |  | 0.91 | 0.83 | 0.93 | 0.88 | 0.85 |
| Specificity  (micro) | 0.89 | 0.86 | 0.92 | 0.91 | 0.85 |  | 0.91 | 0.88 | 0.94 | 0.92 | 0.87 |
| F1 score  (macro) | 0.65 | 0.56 | 0.73 | 0.63 | 0.54 |  | 0.69 | 0.58 | 0.77 | 0.57 | 0.57 |
| F1 score (micro) | 0.79 | 0.72 | 0.83 | 0.82 | 0.70 |  | 0.83 | 0.76 | 0.89 | 0.85 | 0.74 |
| AUC (macro) | 0.88 | 0.82 | 0.91 | 0.87 | 0.77 |  | 0.91 | 0.87 | 0.96 | 0.91 | 0.85 |
| AUC (micro) | 0.92 | 0.88 | 0.94 | 0.94 | 0.86 |  | 0.94 | 0.93 | 0.98 | 0.95 | 0.91 |
| **with information of high myopia only** | | | | | | | | | | | |
| Accuracy | 0.78 | 0.74 | 0.77 | 0.79 | 0.70 |  | 0.83 | 0.81 | 0.80 | 0.82 | 0.70 |
| Precision  (macro) | 0.66 | 0.64 | 0.68 | 0.62 | 0.57 |  | 0.72 | 0.74 | 0.66 | 0.59 | 0.57 |
| Precision  (micro) | 0.78 | 0.74 | 0.77 | 0.79 | 0.70 |  | 0.83 | 0.81 | 0.80 | 0.82 | 0.70 |
| Sensitivity  (macro) | 0.71 | 0.69 | 0.69 | 0.60 | 0.56 |  | 0.76 | 0.77 | 0.68 | 0.56 | 0.58 |
| Sensitivity  (micro) | 0.78 | 0.74 | 0.77 | 0.79 | 0.70 |  | 0.83 | 0.81 | 0.80 | 0.82 | 0.70 |
| Specificity  (macro) | 0.88 | 0.87 | 0.88 | 0.87 | 0.82 |  | 0.91 | 0.89 | 0.91 | 0.88 | 0.82 |
| Specificity  (micro) | 0.89 | 0.87 | 0.88 | 0.89 | 0.85 |  | 0.91 | 0.91 | 0.90 | 0.91 | 0.85 |
| F1 score  (macro) | 0.68 | 0.66 | 0.68 | 0.60 | 0.56 |  | 0.73 | 0.75 | 0.66 | 0.57 | 0.58 |
| F1 score (micro) | 0.78 | 0.74 | 0.77 | 0.79 | 0.70 |  | 0.83 | 0.81 | 0.80 | 0.82 | 0.70 |
| AUC (macro) | 0.89 | 0.86 | 0.88 | 0.87 | 0.77 |  | 0.94 | 0.90 | 0.94 | 0.91 | 0.83 |
| AUC (micro) | 0.91 | 0.86 | 0.92 | 0.93 | 0.82 |  | 0.94 | 0.93 | 0.95 | 0.95 | 0.87 |
| **with age, gender, and high myopia information** | | | | | | | | | | | |
| Accuracy | 0.77 | 0.75 | 0.76 | 0.81 | 0.68 |  | 0.83 | 0.80 | 0.79 | 0.83 | 0.79 |
| Precision  (macro) | 0.64 | 0.65 | 0.66 | 0.65 | 0.56 |  | 0.71 | 0.69 | 0.66 | 0.54 | 0.66 |
| Precision  (micro) | 0.77 | 0.75 | 0.76 | 0.81 | 0.68 |  | 0.83 | 0.80 | 0.79 | 0.83 | 0.79 |
| Sensitivity  (macro) | 0.65 | 0.68 | 0.67 | 0.62 | 0.56 |  | 0.72 | 0.73 | 0.70 | 0.58 | 0.65 |
| Sensitivity  (micro) | 0.77 | 0.75 | 0.76 | 0.81 | 0.68 |  | 0.83 | 0.80 | 0.79 | 0.83 | 0.79 |
| Specificity  (macro) | 0.87 | 0.87 | 0.88 | 0.86 | 0.82 |  | 0.91 | 0.89 | 0.89 | 0.86 | 0.88 |
| Specificity  (micro) | 0.89 | 0.87 | 0.88 | 0.90 | 0.84 |  | 0.91 | 0.90 | 0.89 | 0.92 | 0.89 |
| F1 score  (macro) | 0.64 | 0.66 | 0.66 | 0.63 | 0.56 |  | 0.71 | 0.70 | 0.67 | 0.56 | 0.65 |
| F1 score (micro) | 0.77 | 0.75 | 0.76 | 0.81 | 0.68 |  | 0.83 | 0.80 | 0.79 | 0.83 | 0.79 |
| AUC (macro) | 0.89 | 0.88 | 0.88 | 0.92 | 0.78 |  | 0.92 | 0.93 | 0.94 | 0.95 | 0.85 |
| AUC (micro) | 0.93 | 0.90 | 0.91 | 0.94 | 0.83 |  | 0.95 | 0.94 | 0.95 | 0.96 | 0.89 |

AUC, area under receiver operating characteristic curve
